# Pituitary adenylate cyclase-activating polypeptide (PACAP) in zebrafish models of nephrotic syndrome

**DOI:** 10.1371/journal.pone.0182100

**Published:** 2017-07-31

**Authors:** Benedicte Eneman, Mohamed A. Elmonem, Lambertus P. van den Heuvel, Laleh Khodaparast, Ladan Khodaparast, Chris van Geet, Kathleen Freson, Elena Levtchenko

**Affiliations:** 1 Department of Pediatric Nephrology & Growth and Regeneration, University Hospitals Leuven, KU Leuven – University of Leuven, Leuven, Belgium; 2 Department of Clinical and Chemical Pathology, Faculty of Medicine, Cairo University, Cairo, Egypt; 3 Department of Pediatric Nephrology, Radboud University Medical Center, Nijmegen, the Netherlands; 4 Department of Cellular and Molecular Medicine, Switch Laboratory, VIB, University Hospitals Leuven, KU Leuven – University of Leuven, Leuven, Belgium; 5 Department of Cardiovascular Sciences, Center for Molecular and Vascular Biology, KU Leuven - University of Leuven, Leuven, Belgium; Universite de Rouen, FRANCE

## Abstract

Pituitary adenylate cyclase-activating polypeptide (PACAP) is an inhibitor of megakaryopoiesis and platelet function. Recently, PACAP deficiency was observed in children with nephrotic syndrome (NS), associated with increased platelet count and aggregability and increased risk of thrombosis. To further study PACAP deficiency in NS, we used transgenic *Tg(cd41*:*EGFP)* zebrafish with GFP-labeled thrombocytes. We generated two models for congenital NS, a morpholino injected model targeting *nphs1* (nephrin), which is mutated in the Finnish-type congenital NS. The second model was induced by exposure to the nephrotoxic compound adriamycin. Nephrin RNA expression was quantified and zebrafish embryos were live-screened for proteinuria and pericardial edema as evidence of renal impairment. Protein levels of PACAP and its binding-protein ceruloplasmin were measured and GFP-labeled thrombocytes were quantified. We also evaluated the effects of PACAP morpholino injection and the rescue effects of PACAP-38 peptide in both congenital NS models. Nephrin downregulation and pericardial edema were observed in both nephrin morpholino injected and adriamycin exposed congenital NS models. However, PACAP deficiency was demonstrated only in the adriamycin exposed condition. Ceruloplasmin levels and the number of GFP-labeled thrombocytes remained unchanged in both models. PACAP morpholino injections worsened survival rates and the edema phenotype in both congenital NS models while injection with human PACAP-38 could only rescue the adriamycin exposed model. We hereby report, for the first time, PACAP deficiency in a NS zebrafish model as a consequence of adriamycin exposure. However, distinct from the human congenital NS, both zebrafish models retained normal levels of ceruloplasmin and thrombocytes. We further extend the renoprotective effects of the PACAP-38 peptide against adriamycin toxicity in zebrafish.

## Introduction

Nephrotic syndrome (NS) is a disease of the glomerular filtration barrier, characterized by severe proteinuria, hypoalbuminemia, edema, and hyperlipidemia [1]. Congenital NS (CNS) is a severe form of NS, manifesting within 3 months of life with profound edema and proteinuria being present intrauterine. CNS is rarely the result of non-genetic causes, such as intrauterine infections, but most frequently, it is caused by genetic mutations in one of the following genes: *NPHS1*, *NPHS2*, *WT1*, *LAMB2* or *PLCE1* [2,3]. *NPHS1* encodes for nephrin, a structural component of the slit diaphragm [4], which plays a role in intracellular signaling and interacts with the podocyte cytoskeleton [5]. *NPHS1* mutations and subsequent disruption of the slit diaphragm cause CNS of the Finnish-type, a common and severe form of CNS with extremely pronounced proteinuria.

Patients with NS have an increased risk of thromboembolic events, both deep venous and arterial thrombosis [6,7]. Elevated levels of prothrombotic factors such as factor V, factor VIII, von Willebrand factor and fibrinogen, and decreased levels of antithrombotic factors such as antithrombin III, protein C and protein S were described in NS patients and probably underlie the increased risk of deep venous thrombosis [6]. However, the fact that the risk of both venous and arterial thrombosis is elevated in NS, points to an additional role for blood platelets. Indeed, increased platelet counts and hyperaggregability were repeatedly observed in NS patients [8].

Recently, we found evidence that deficiency of pituitary adenylate cyclase activating polypeptide (PACAP) plays a role in the increased platelet count and hyperaggregability in CNS [9]. PACAP is a highly conserved neuropeptide [10,11]. In human plasma, PACAP is bound to its carrier protein ceruloplasmin (132 kDa), which prevents it from rapid degradation [12]. PACAP is widely expressed in the nervous system, but also in several peripheral tissues, where it takes part in diverse biological processes [11]. PACAP can bind three different G-protein-coupled receptors: the PAC1 receptor, which is PACAP specific, and the VPAC1 and VPAC2 receptors, which bind both vasoactive intestinal peptide (VIP) and PACAP and are coupled to adenylyl cyclase [13–15]. Interestingly, PACAP was identified as an inhibitor of megakaryopoiesis and platelet activation. Freson *et al* studied patients with elevated PACAP plasma levels due to a partial trisomy 18p and transgenic mice with megakaryocyte-specific PACAP overexpression [16,17]. They found that PACAP inhibits megakaryopoiesis and platelet function and prolongs the bleeding time, via activation of adenylyl cyclase-coupled VPAC1 receptors on megakaryocytes and platelets.

The opposite phenotype with stimulation of platelet function and megakaryopoiesis was obtained in wild-type mice injected with a PACAP antagonist PACAP(6–38) or a monoclonal inhibitory antibody against PACAP or its receptor VPAC1, as well as in PACAP knockout mice. Peeters *et al* further showed a thrombopoietic effect of VPAC1 inhibition in thrombocytopenia murine models [18]. We recently reported urinary losses of PACAP and ceruloplasmin in NS children, which was more pronounced in children with the CNS of the Finnish type than in children with idiopathic NS (INS); however, platelet hyperaggregability during nephrotic state was equally present [9]. We further confirmed that megakaryopoiesis and platelet aggregability were increased in CNS patients during the nephrotic state in comparison to the non-nephrotic state after bilateral nephrectomy and that this effect could be reversed by the addition of recombinant PACAP-38 [9].

To support our hypothesis that PACAP deficiency is the cause of increased megakaryopoiesis in CNS, and to further study the mechanisms behind this effect, we sought a NS animal model. Constitutive nephrin knockout mice are not available as the animals die within 24 hours after birth [19]. Zebrafish larvae are currently widely used in kidney research as the structure of the glomerular filtration barrier is similar to the mammalian filter, with the presence of fenestrated endothelial cells, a glomerular basement membrane and podocytes with a slit diaphragm. Moreover, in zebrafish, a complete maturation of the glomerulus is observed within 3 days post fertilization (3 dpf) [20].

For our study, we used two previously characterized NS zebrafish models [21,22]. The first was obtained by injection of a morpholino targeting nephrin (*nphs1)* [21]. Nephrin depleted zebrafish were previously shown to have podocyte foot processes effacement and absent slit diaphragms, in accordance with observations for Finnish-type CNS in humans. Moreover, due to loss of the podocyte barrier function, nephrin depleted embryos developed a phenotype of proteinuria and pericardial edema at 4 dpf [21]. The second NS zebrafish model was obtained by exposure to low concentrations of adriamycin [22]. Adriamycin exposure leads to podocyte developmental defects and decreased nephrin expression, with subsequent functional impairment of the glomerular filtration barrier, causing proteinuria and pericardial edema, similar to the observations for human CNS. We established these two NS models in *Tg(cd41*:*EGFP)* transgenic zebrafish with GFP-labeled thrombocytes, previously used to study thrombocyte formation [23]. The aim of this study was to investigate whether these CNS zebrafish models develop PACAP deficiency and increased thrombocyte numbers as found in human CNS.

## Material and methods

### Embryo collection

All zebrafish experiments and protocols were approved by the Ethical Committee of the Catholic University of Leuven. *Tg(cd41*:*EGFP)* transgenic zebrafish embryos were a gift from Dr. L. Zon (Hematology Division, Brigham and Women's Hospital's, Boston, MA, USA). Embryos were kept at 28.5°C in egg water medium (Instant Ocean Sea Salts, 60 μg/ml, and methylene blue, 0.3 ppm).

### Morpholino injection

*Tg(cd41*:*EGFP)* transgenic *Danio rerio* embryos [23] were injected at the one-cell stage with an exon splice donor sites targeting morpholino for nephrin (5’-CGCTGTCCATTACCTTTCAGGCTCC-3’) at 50, 100 and 200 μM, as previously described^22^. The morpholino was diluted in an injection solution (0.5% Phenol red (Sigma, St-Louis, MO, USA) 1/10 in NaCl 0.9% (v/v)). Off-target effects were excluded by the inclusion of standard control morpholino (5’- CCTCTTACCTCAGTTACAATTTATA-3’) injected embryos. Since PACAP is translated by two genes in zebrafish (*adcyap1a* and *adcyap1b*), we injected splicing morpholinos for both genes together in the PACAP suppression experiments (*adcyap1a*: 5’- CCTCCTCTGCGTTAGAGAAATAGGA-3’ and *adcyap1b*: 5’- CCTCCGCTGCAAATATAGGAACTAT-3’). PACAP, nephrin, and control morpholinos were all obtained from Gene-Tools LLC (Philomath, OR, USA).

### Embryo exposure to adriamycin

*Tg(cd41*:*EGFP)* transgenic zebrafish embryos [23] were incubated in either free medium, 10 or 30 μM of adriamycin (Sigma) starting from 9 hpf as previously described [22]. Adriamycin was removed from the medium after 48 hours (at 57 hpf). Embryos were next washed several times with medium and further immersed in the adriamycin-free medium. PACAP rescue experiments for both CNS models were performed through the injection of recombinant human PACAP-38 (Bachem, Bubendorf, Switzerland). The human peptide has 85% identity and 94% similarity with zebrafish PACAP (S1 Fig).

### Phenotype characterization

Fluorescent microscopy was used for live-screening of embryos/larvae at 24, 48, 72, 96 and 120 hpf. The number of thrombocytes in the caudal hematopoietic tissue (CHT) of zebrafish embryos/larvae was recorded through the detection of GFP-labeled thrombocytes. Images were captured with a Zeiss Axiocam HR camera using AxioVision software 4.8 (Carl Zeiss, Jena, Germany). Pixel intensity of fluorescent pictures was measured using ImageJ software (http://rsbweb.nih.gov/ij/).

### Evaluation of glomerular blood filtration

A qualitative assay was performed to evaluate glomerular blood filtration, as previously described [24]. Rhodamine-labeled 70 kDa-dextran (2 nl of 50 mg/ml) (Sigma) was injected into the cardiac venous sinus of 75 hpf old larvae. Images were captured 5 hours after injection using a Zeiss Axiocam HR camera and AxioVision software 4.8 (Carl Zeiss). Fluorescence intensity in the retinal vascular bed was measured using ImageJ software.

### RNA isolation, reverse transcription PCR, and quantitative real-time PCR

Total RNA was extracted from at least 15 zebrafish embryos per sample at various stages of development using Trizol Reagent (Invitrogen, Waltham, USA). RNA concentration was measured using Nanodrop 2000c (Thermo Scientific, Waltham, USA). cDNA was produced from 1 μg of RNA using SuperScriptR III reverse transcriptase (Life Technologies, Carlsbad, USA), oligo (dT) primers, random hexamer primers and dNTP mix (Invitrogen). Pixel intensities of reverse transcription PCR (RT-PCR) bands were measured using ImageJ software. Quantitative real-time PCR (qPCR) was performed with Platinum SYBR Green qPCR mix (Invitrogen). Sequences of forward and reverse primers were: *nphs1* (Fw) 5’-GCAAGCTACATGTATGTAGACGTGT-3’ and *nphs1* (Rv) 5’-TCCTGTGAAATGCTGCTGGTGTC-3’, *adcyap1a* (Fw) 5’-CGCCTCTGAGTTACCCGAAAA-3’ and *adcyap1a* (Rv) 5’-TAGCGAGCCGCCGTCCTTTG-3’, *adcyap1b* (Fw) 5’-TCAGGGAAGAGGTGCTGTGAGGA-3’ and *adcyap1b* (Rv) 5’-CATCTGTTTTCGGTAGCGACTGT-3’, *vip* (Fw) 5’-GGCTCTTCACAAGCGGATAC-3’ and *vip* (Rv) 5’-ATCATCACTGACCCGCTTTC-3’. As a reference gene, we used Eukaryotic translation elongation factor 1 alpha 1, like 1 *(eef1a1l1)* (Fw) 5’-CTTCTCAGGCTGACTGTGC-3’ and *eef1a1l1* (Rv) 5’-CCGCTAGCATTACCCTCC-3’ [25].

### Immunoblot analysis

Protein lysates were obtained from control versus nephrin morphants and from adriamycin non-exposed versus exposed zebrafish larvae. Blots were incubated with a rabbit polyclonal anti-PACAP antibody (produced in our laboratory, as previously described) [16], a rabbit monoclonal anti-ceruloplasmin antibody (Dako, Glostrup, Denmark), a goat polyclonal anti-GFP antibody (Rockland Immunochemicals, Gilbertsville, USA) and a rabbit monoclonal anti-β-actin antibody (Cell Signaling Technology, Boston, USA). Secondary anti-rabbit and anti-goat antibodies were from Dako. Blots were stained with Western blotting electrochemiluminescence (ECL) detection reagent (Thermo Scientific, Rockford, USA). Detection of signal and measuring of band intensity were performed with ImageJ software. The band intensities of PACAP, ceruloplasmin, and GFP were corrected for the band intensities of the loading control β-actin [26] and were expressed as a percentage of the control condition. All Western blots were performed at multiple time points during the first 6 days of development and at least in duplicate.

### Flow cytometric analysis of CD41 positive thrombocytes

Control versus nephrin morpholino injected *Tg(cd41*:*EGFP)* transgenic embryos were dechorionated if not hatched at 72 hpf, and digested with 0.02% trypsin/EDTA for 15 minutes at 28°C. Each sample was pipetted 10 times up and down, for getting a single cell suspension. Cells were filtered through a 40 μm cell strainer and centrifuged (770 *g* for 10 minutes at room temperature). Pellets were then resuspended in phosphate buffered saline (PBS). The Cell Diva software was used for two-color immunofluorescence acquisition on a FACSCanto II flow cytometer (BD Biosciences, San Diego, USA) and for data analysis determining the percentage of GFP-positive thrombocytes.

### Statistical analysis

Statistical analysis was performed by the SPSS Statistics software, ver. 22 (IBM, Armonk, New York, USA). Continuous variables were compared using two-tailed Student’s t-test for unpaired data. P < 0.05 was regarded as statistically significant.

## Results

### Nephrin depleted CNS zebrafish model

**Fig 1:**
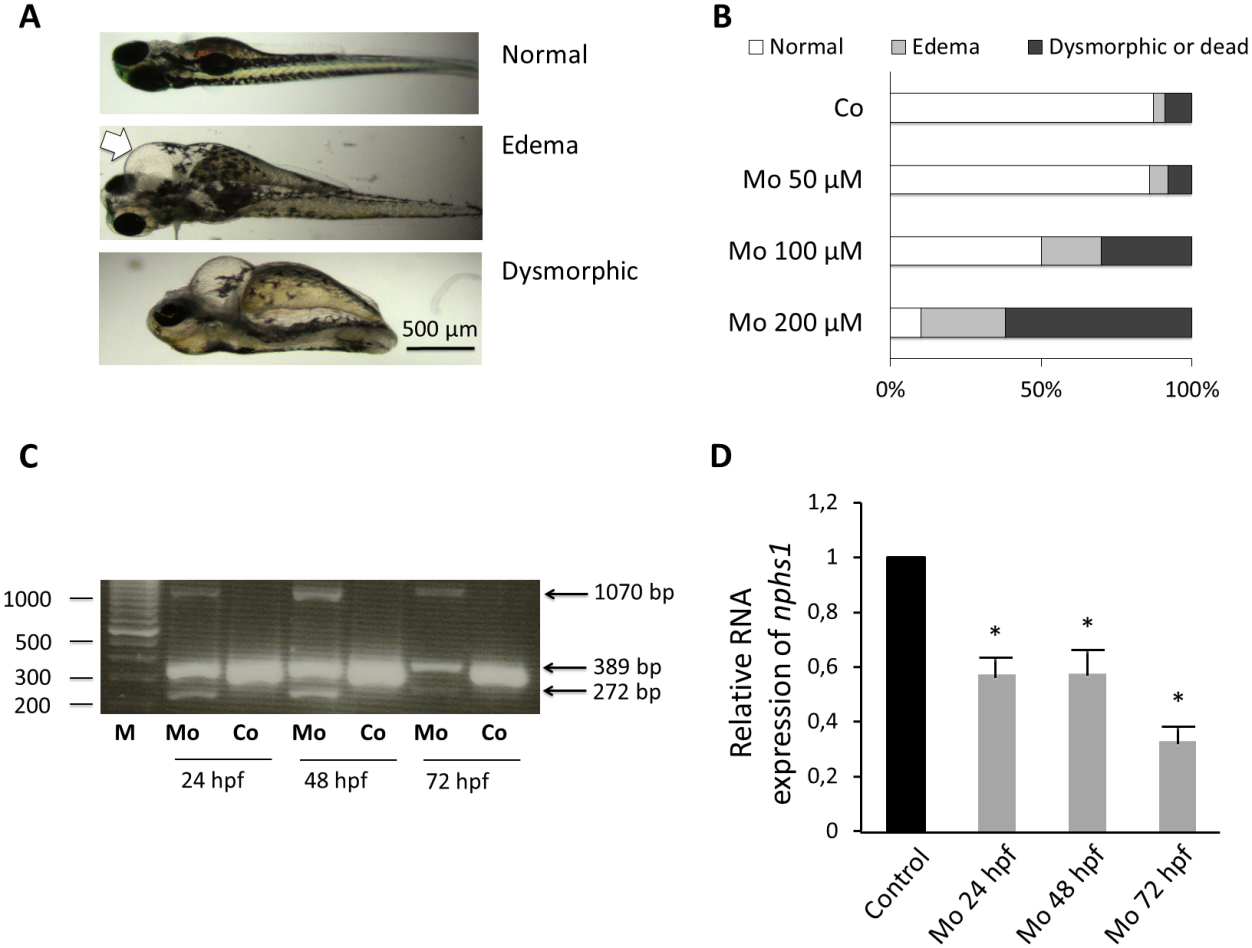
Phenotype and nephrin expression in nephrin depleted zebrafish. (A) Three categories of phenotypes were defined in nephrin morpholino injected embryos: embryos without edema (normal), embryos with visible pericardial edema (white arrow) and dysmorphic or dead embryos. Pictures were taken at 5 dpf. (B) 80 embryos per condition were live-screened at 5 dpf and assigned to a phenotype category. Increasing concentrations of the injected nephrin morpholino were associated with an increasing percentage of embryos with pericardial edema, but also with increased numbers of severely dysmorphic or dead embryos. (C) RT-PCR was performed using total RNA extracted from nephrin morpholino (100 μM) and control morpholino injected embryos at 24, 48 and 72 hpf. Reduced expression of normal nephrin (389 bp) was found in the nephrin depleted versus control embryos. Moreover, injection of nephrin morpholino induced alternative splicing resulted in 272 and 1070 bp fragments, resulting from an exon deletion and a retained intron, respectively. (D) Quantitation of *nphs1* RNA expression in control versus nephrin morpholino injected larvae after 24, 48 and 72 h of injection. M, marker; Mo, nephrin morpholino injected; Co, control morpholino injected.

The first CNS zebrafish model was obtained by injection of *Tg(cd41*:*EGFP)* transgenic zebrafish with a splice site morpholino targeting *nphs1* gene [21]. The zebrafish embryos were live-screened for the nephrotic phenotype of pericardial edema at 3 and 5 dpf and were divided into 3 different categories: embryos with a normal phenotype, embryos with visible pericardial edema and severe dysmorphic or dead embryos (Fig 1A). A phenotype of pericardial edema was obtained at 5 dpf in only 6, 20 and 28% of embryos after nephrin morpholino injections of 50, 100 and 200 μM, respectively (Fig 1B). Phenotypes at 3 dpf were basically similar to those observed at 5 dpf. For increasing concentrations of nephrin morpholino, an increasing percentage of embryos showed a severely dysmorphic phenotype or died. All further experiments were performed with 100 μM nephrin morpholino. RT-PCR was performed using total RNA extracted from nephrin morpholino and control morpholino zebrafish at 24, 48 and 72 hpf. Reduced nephrin expression (389 bp) was seen in RNA samples extracted from nephrin morphants compared to control embryos (Fig 1C). Quantification of nephrin expression in nephrin morphants was 57.2, 57.6 and 33.0% compared to controls at 24, 48 and 72 hpf, respectively, P < 0.05 (Fig 1D). Abnormal RT-PCR bands of 272 and 1070 bp were only detected for the splice nephrin morpholino injected embryos resulting from exon deletion and retention of an adjacent intron, respectively.

Nephrin depleted embryos showed significant downregulation of *adcyap1a* after 48 and 72 hpf compared to control morpholino, while the second PACAP translating gene (*adcyap1b*) showed no significant difference (S2 Fig). Since both VIP and PACAP act on the VPAC1 receptors with a similar affinity [27], we also tested for the expression of the zebrafish *vip* gene using the same samples and detected its downregulation at 48 and 72 hpf similar to *adcyap1a* (S3 Fig). All three genes tend to be upregulated normally during the first three days of development.

**Fig 2:**
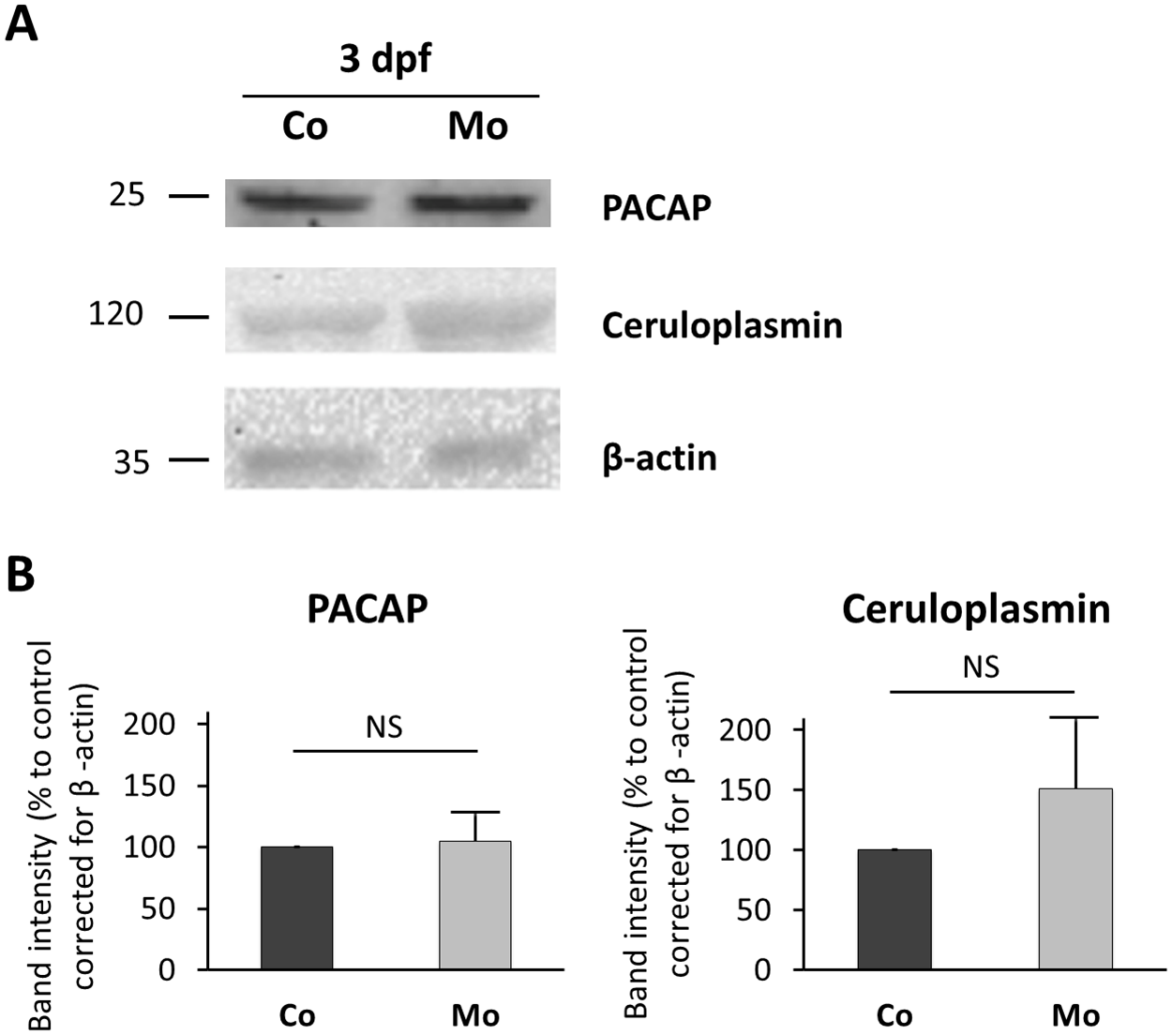
PACAP and ceruloplasmin protein levels in nephrin depleted zebrafish. (A) Western blots for PACAP, ceruloplasmin, and β-actin (loading control) were performed using whole zebrafish lysates at 3 dpf for nephrin depleted (100 μM Nephrin morpholino) compared to control embryos. (B) Signal intensity of Western blot bands was measured using ImageJ software. Graphs represent means ± SD from two repeated experiments. A representative blot is shown. Means were compared using a two-tailed unpaired Student *t* test, but no significant difference was observed. Mo, nephrin morpholino injected; Co, control morpholino injected.

Nephrin depleted embryos with visible pericardial edema were selected to further study PACAP and ceruloplasmin expression using Western blot analysis of total embryo lysates. No differences in PACAP levels were observed in nephrin depleted versus controls embryos (Fig 2). Western blot for ceruloplasmin also showed no significant difference in nephrin depleted and control embryos at 3 dpf. Western blots for PACAP and ceruloplasmin were repeated at 5 dpf with similar results.

**Fig 3:**
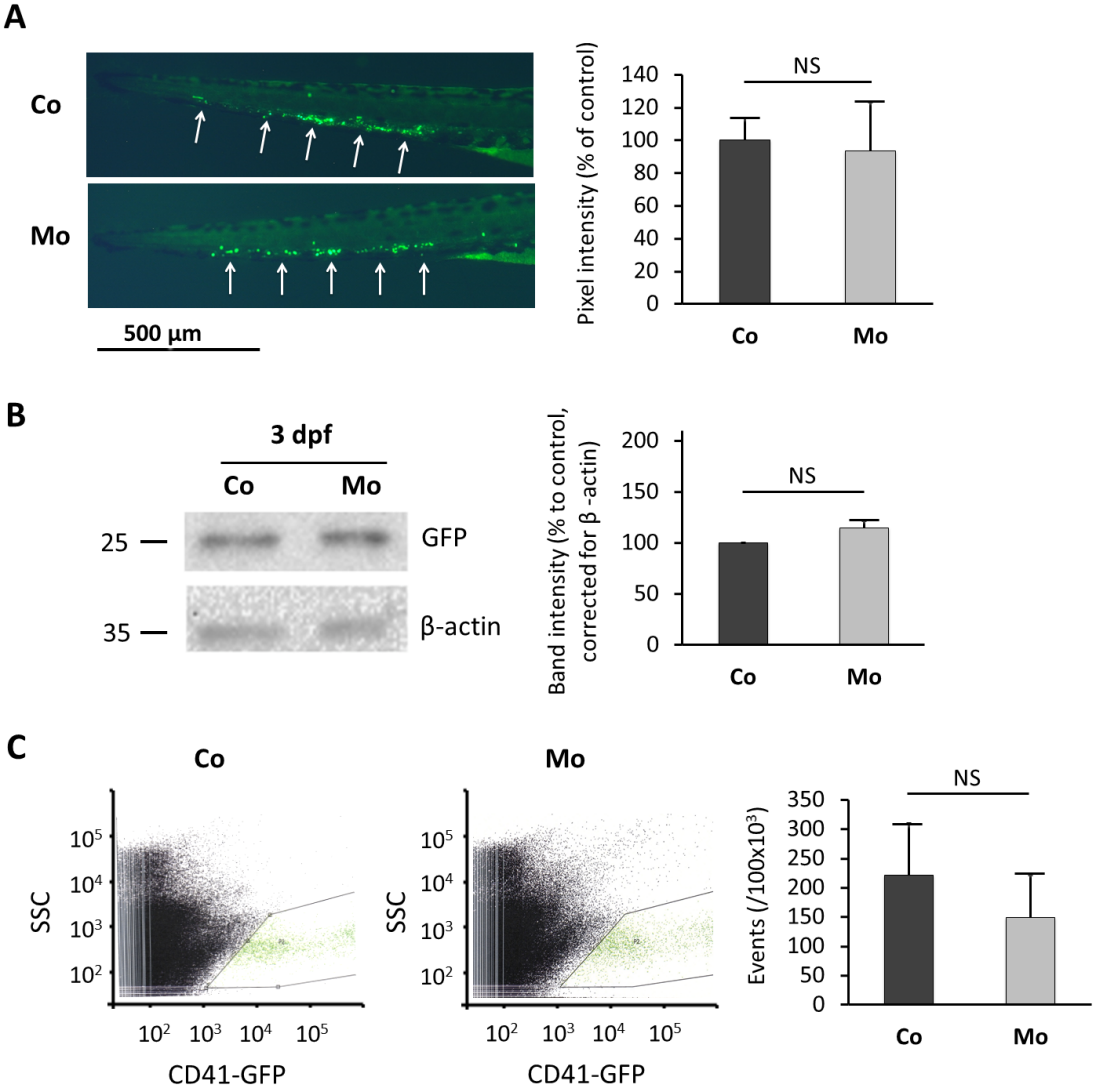
Quantification of thrombocytes in nephrin depleted *Tg(cd41*:*EGFP)* transgenic zebrafish. (A) LEFT: GFP-labeled thrombocytes and thrombocyte precursors are formed in the zebrafish caudal hematopoietic tissue (CHT) region (white arrows) at 3 dpf. Representative pictures of the CHT region of a control and a nephrin depleted (100 μM nephrin morpholino) embryo at 3 dpf are shown. No obvious differences in thrombocyte numbers were observed. RIGHT: Pixel intensity was measured using ImageJ software. The graph represents means ± SD from measurements in three embryos per condition. (B) LEFT: Western blot for GFP and β-actin (loading control) was performed using total zebrafish lysates at 3 dpf. A representative blot is shown. RIGHT: Signal intensity was measured using ImageJ software. The graph represents means ± SD from measurements in two repeated experiments. (C) LEFT: Fluorescence-activated cell sorter (FACS) analysis of control zebrafish lysates for CD41 positive cells was performed at 3 dpf. MIDDLE: FACS analysis of morphant zebrafish lysates for CD41 positive cells was performed at 3 dpf. RIGHT: A diagrammatic representation of the number of GFP-positive cells per 100,000 counted cells. For each zebrafish lysate, 500,000 cells were counted and analyzed. Graphs represent means ± SD from three repeated experiments performed in duplicate. Mo, nephrin morpholino injected; Co, control morpholino injected; SCC, side scatter; GFP, green fluorescent protein.

*Tg(cd41*:*EGFP)* transgenic zebrafish were next used to study the effect of nephrin depletion on thrombocyte formation. Fluorescence microscopy did not show a difference between GFP-labeled thrombocytes in nephrin morpholino injected (100 μM) versus control embryos at 3 dpf (Fig 3A). Quantification of GFP expression via Western blot analysis also revealed no differences in thrombocyte numbers (Fig 3B). Finally, flow cytometric analysis of lysates from whole zebrafish embryos did not show any differences in the number of GFP-positive thrombocytes (Fig 3C). Western blot and flow cytometry were repeated at 5 dpf with similar results.

**Fig 4:**
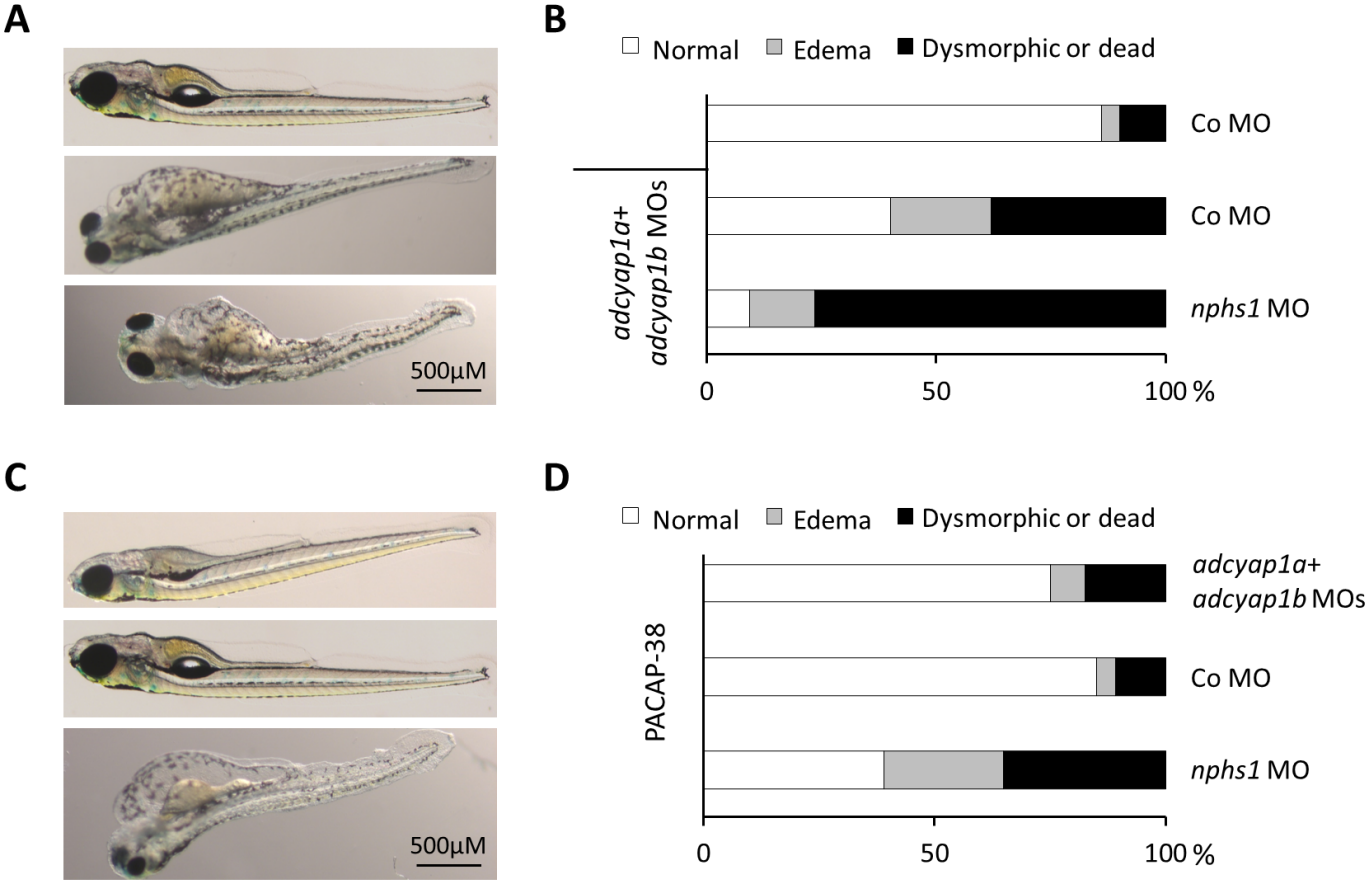
PACAP morpholino suppression and PACAP-38 rescue in nephrin depleted embryos. (A) Representative larvae of the phenotypes observed in different groups with or without PACAP (*adcyap1a and adcyap1b*) morpholinos injections (100 μM each). (B) Approximately 100 injected embryos per condition were live-screened at 4 dpf and assigned to a phenotype category. Compared to the control morpholino injection alone, PACAP morpholinos produced a harmful effect when injected together with the control morpholino and a devastating effect with the *nphs1* morpholino. (C) Representative larvae of the phenotypes observed in different groups with human PACAP-38 injection (5μM). (D) Approximately 100 injected embryos per condition were live-screened at 4 dpf and assigned to a phenotype category. Human PACAP-38 could rescue to a great extent the PACAP morpholino injected embryos; however, they produced no beneficial effect on the *nphs1* morpholino injected embryos.

In order to evaluate if PACAP could have an effect on the phenotype of nephrin depleted embryos, we conducted independent series of experiments through the injection of zebrafish PACAP morpholinos (*adcyap1a and adcyap1b*) in embryos with either *nphs1* morpholino or control morpholino injected (approximately 100 embryos per group). As seen in Fig 4, co-injection of both PACAP morpholinos had a harmful effect compared to control morpholino-injected embryos and a devastating effect on *nphs1* morpholino injected embryos (Fig 4A and 4B). On the other hand, human PACAP-38 could rescue the phenotype of embryos injected with *adcyap1a and adcyap1b* morpholinos, but not of those injected with *nphs1* morpholino (Fig 4C and 4D).

### Adriamycin exposed CNS zebrafish model

**Fig 5:**
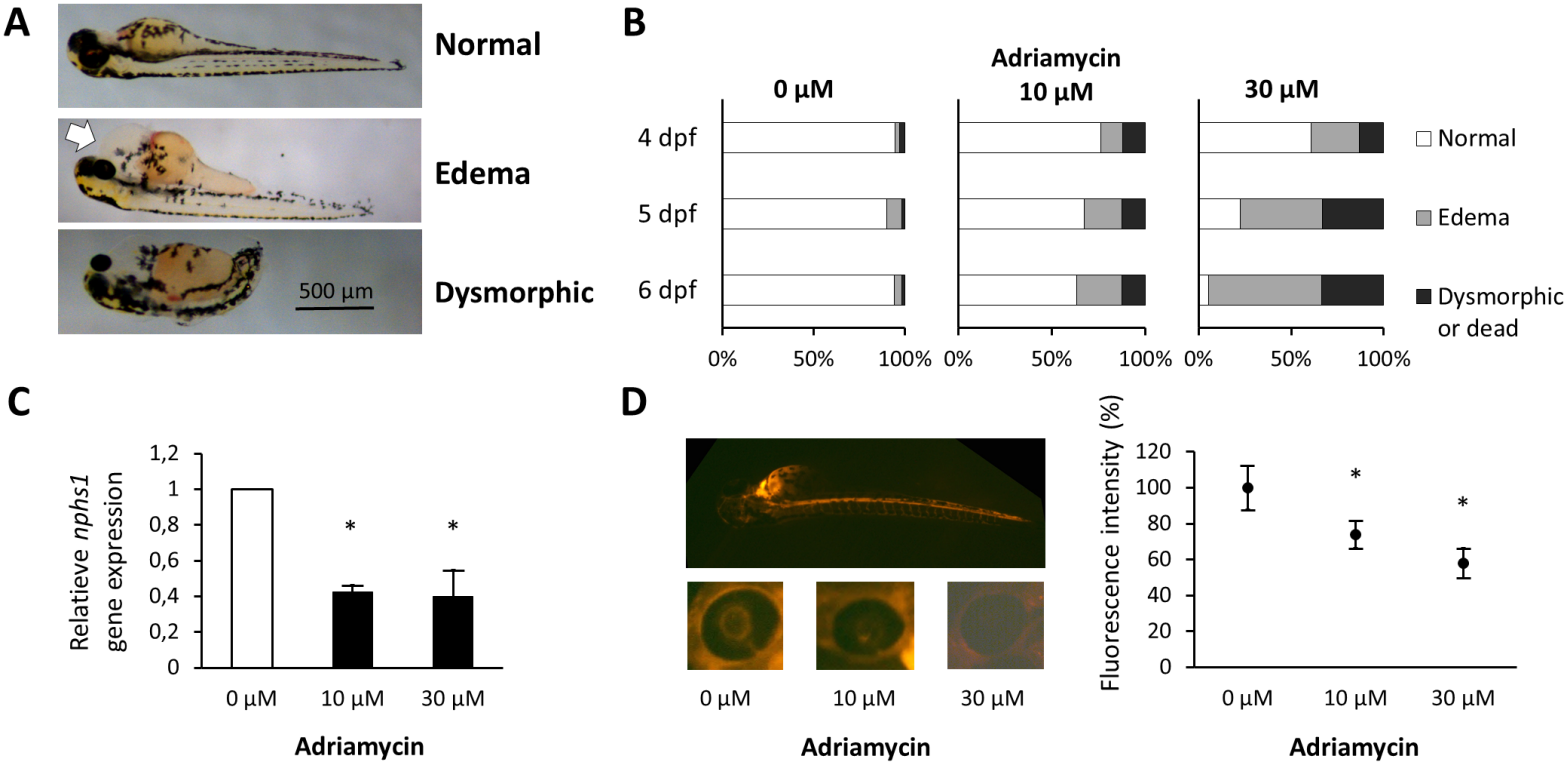
Phenotype analysis, nephrin expression and evaluation of glomerular function in adriamycin exposed zebrafish. (A) Different categories of phenotype were defined in the adriamycin exposed embryos at 4 dpf: embryos with a normal phenotype, embryos with visible pericardial edema (white arrow) and dysmorphic or dead embryos. (B) 100 embryos per condition were live-screened at 4 dpf and assigned to a phenotype category. Increasing adriamycin concentrations in the culture medium were associated with an increasing percentage of embryos with pericardial edema. (C) qPCR was performed using total RNA from control and adriamycin exposed embryos at 4 dpf. A significantly decreased expression of nephrin (corrected for housekeeping gene *elfa*) was observed in the adriamycin exposed embryos compared to the control fish. The experiment was performed twice in triplicate. Bars represent means ± SD. * P<0.05 in comparison to condition without the addition of adriamycin. (D) Rhodamine-labeled 70 kDa dextran was injected in the cardiac venous sinus of 75 hpf old embryos. LEFT: A representative immunofluorescence picture of a control embryo immediately after injection shows the distribution of fluorescence through the vascular system of the zebrafish larva. A dose-dependent diminishing effect of adriamycin on fluorescence recorded in the fish eye 5 hours after injection was observed. Representative images of the eye from 0, 10 and 30 μM adriamycin treated embryos 5 hours after injection are shown. RIGHT: A diagrammatic representation shows the quantification of the mean fluorescence intensity ± SD recorded in the retinal vascular bed. * P < 0.05 in comparison to condition without the addition of adriamycin.

The adriamycin model of NS in zebrafish was previously described [22]. We applied this strategy in *Tg(cd41*:*EGFP)* transgenic zebrafish embryos and exposed embryos at 9 hpf to adriamycin for 48 hours. Given the toxicity of adriamycin, zebrafish embryos were immersed for 40 hours in the adriamycin-free medium after adriamycin exposure, before further manipulation. Hence, all analyses were performed at 4 instead of 3 dpf. The zebrafish embryos were live-screened for the nephrotic phenotype of pericardial edema at 4 dpf (Fig 5A). A dose and time dependent increase in embryos with a phenotype of pericardial edema was observed (Fig 5B). Quantitative PCR (qPCR) showed significantly reduced expression of *nphs1* in adriamycin exposed zebrafish embryos compared to control fish (Fig 5C). An evaluation of the glomerular function was performed by injection of rhodamine-labeled 70 kDa dextran in 75 hpf old embryos. In the adriamycin treated embryos, a statistically significant lower level of fluorescence intensity was detected 5 hours after injection in the retinal vascular bed of injected embryos, indicating increased glomerular permeability and proteinuria (Fig 5D).

Similar to the published zebrafish model due to adriamycin exposure [22], reduced nephrin RNA expression was confirmed in the adriamycin exposed *Tg(cd41*:*EGFP)* embryos. We further evaluated the expression of both PACAP genes at 4, 5 and 6 dpf after exposure to adriamycin. Similar to the nephrin-injected morpholinos, only *adcyap1a* was significantly downregulated upon the high dose of adriamycin (30 μM), while the expression of both *adcyap1b* and *vip* remained unaffected (S2 and S3 Figs).

**Fig 6:**
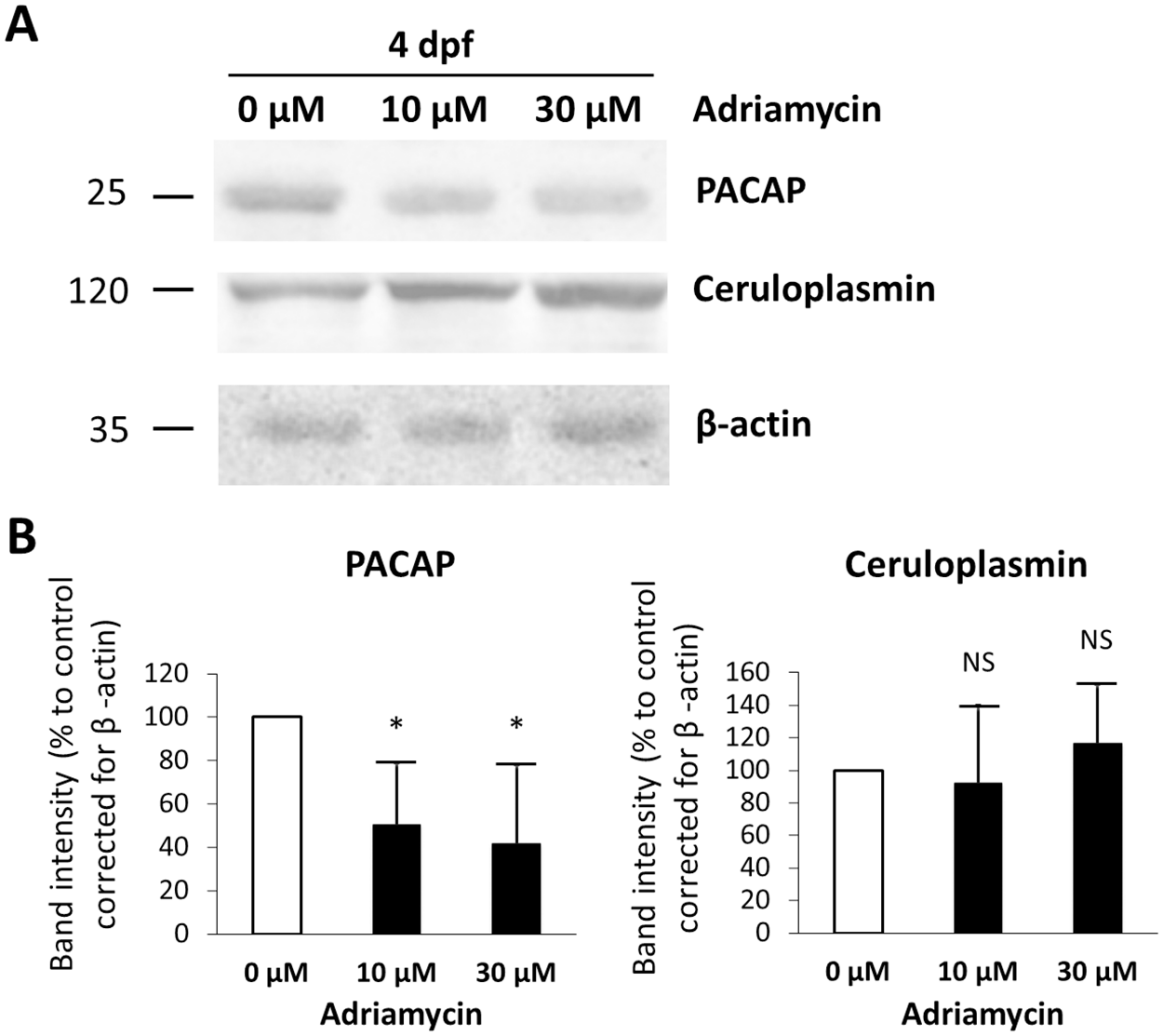
PACAP and ceruloplasmin protein levels in the adriamycin treated zebrafish. (A) Western blot for PACAP, ceruloplasmin, and β-actin (loading control) was performed using total zebrafish lysates. A representative blot is shown. (B) Pixel intensity of Western blot bands was measured using ImageJ software. Graphs represent means ± SD of signal intensity from two repeated experiments. * P < 0.05 (two-tailed unpaired Student *t* test).

This model was further analyzed for PACAP and ceruloplasmin protein levels and thrombocyte formation. Western blot for PACAP was performed using total zebrafish lysates that showed a reduced expression in nephrotic fish compared to controls (P < 0.05) (Fig 6). The signal intensity for ceruloplasmin on Western blot was not significantly different between nephrotic and control embryos (Fig 6).

**Fig 7:**
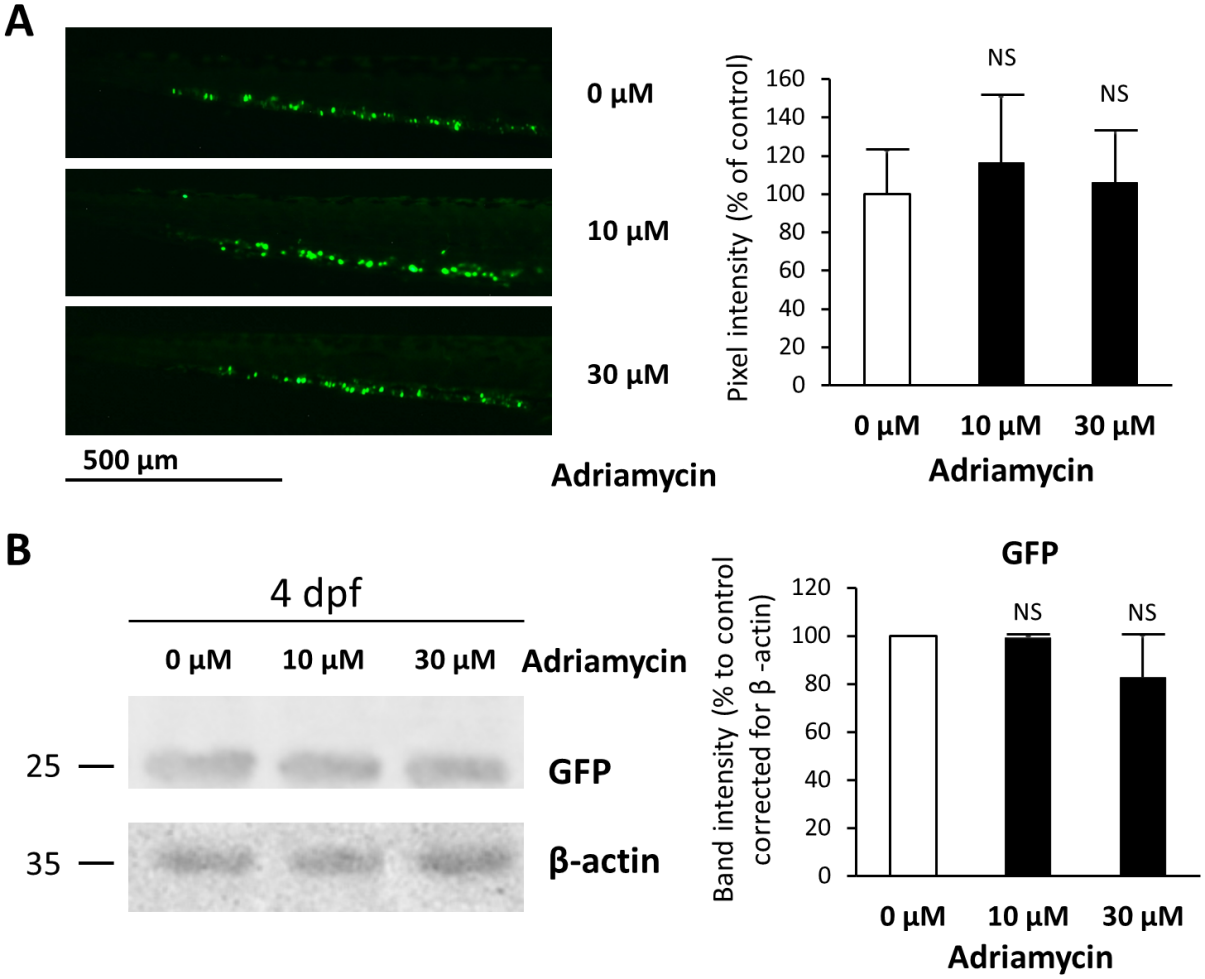
Quantification of thrombocytes in adriamycin treated zebrafish. (A) LEFT: GFP-labeled thrombocytes and thrombocyte precursors are formed in the zebrafish caudal hematopoietic tissue (CHT). Representative pictures of the CHT region at 4 dpf of embryos exposed to adriamycin 0, 10 and 30 μM are shown. No obvious difference in thrombocytes was observed between the controls and the adriamycin exposed fish. RIGHT: Pixel intensity was measured using ImageJ software. The graph represents means ± SD from measurements in three embryos per condition. No significant difference was observed between adriamycin exposed and control embryos. (B) LEFT: Western blot for GFP and β-actin (loading control) was performed using total zebrafish lysates at 4 dpf. A representative blot is shown. RIGHT: Signal intensity was measured using ImageJ software. The graph represents means ± SD from measurements in two repeated experiments. No significant difference was observed between adriamycin exposed and control embryos.

GFP-labeled thrombocytes quantified by fluorescence microscopy revealed no differences after quantification of pixel intensity (Fig 7A) and western blot for GFP expression was similar in all embryo lysates (Fig 7B). Western blots for PACAP, ceruloplasmin, and GFP were repeated at 5 and 6 dpf to investigate a time-dependent effect. However, these experiments remained similar to the data obtained for 4 dpf.

**Fig 8:**
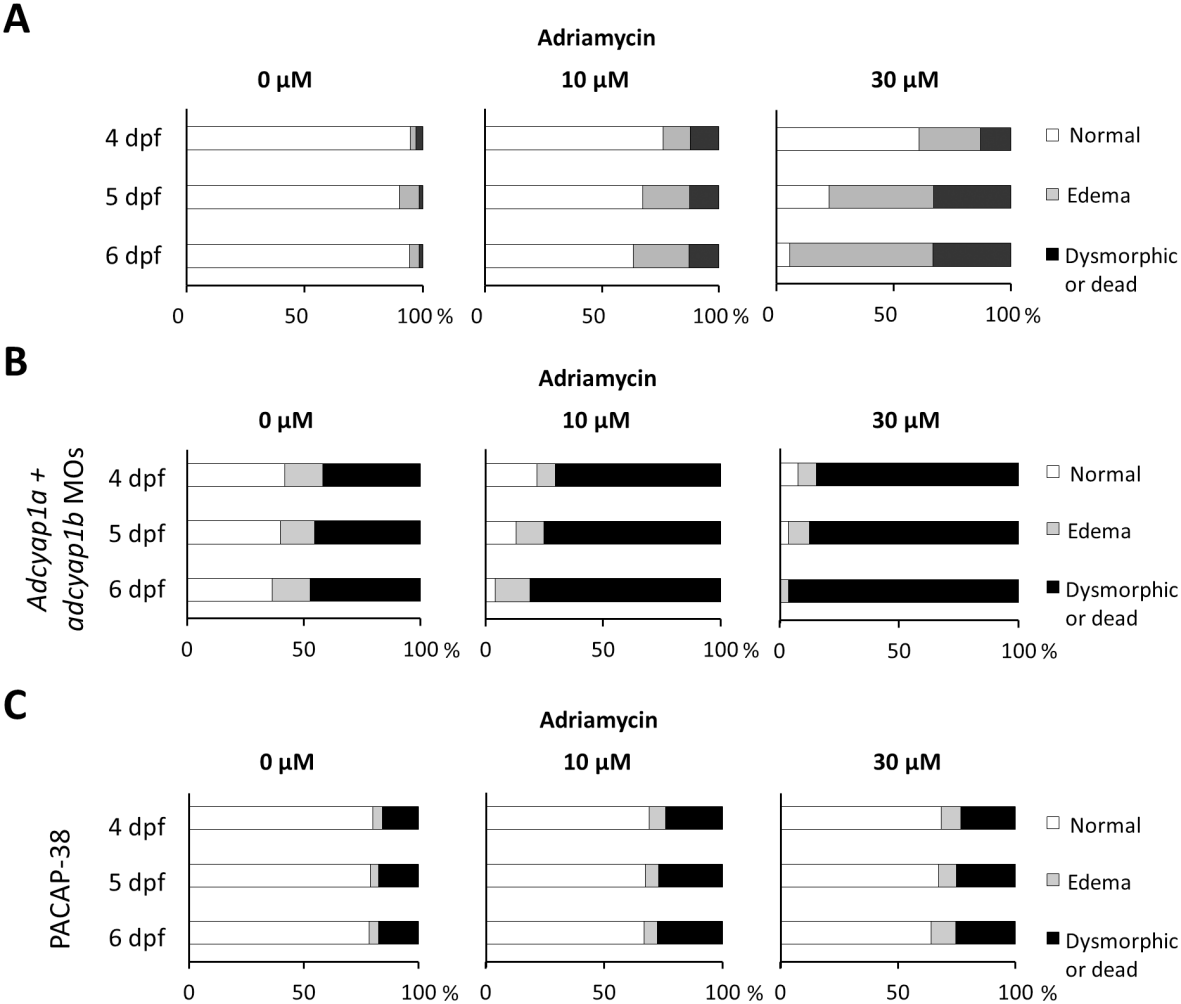
PACAP morpholino suppression and PACAP-38 rescue in adriamycin exposed embryos. (A) Approximately 100 embryos exposed to adriamycin (0, 10 or 30 μM) in the swimming water (no injections), were live-screened at 4, 5 and 6 dpf and assigned to a phenotype category. (B) Approximately 100 embryos injected with PACAP morpholinos (*adcyap1a* and *adcyap1b*, 100 μM) were exposed to adriamycin (0, 10 or 30 μM) and then live-screened at 4, 5 and 6 dpf. PACAP morpholinos had a devastating effect on survival and morphology of the adriamycin exposed embryos. (C) Approximately 100 embryos injected with the human PACAP-38 peptide (5 μM) were exposed to adriamycin (0, 10 or 30 μM) and then live-screened at 4, 5 and 6 dpf. Human PACAP-38 could rescue the nephrotic phenotype in zebrafish with adriamycin exposure especially with the higher concentration (30 μM).

Similar to the nephrin depleted embryos, we evaluated whether PACAP could have an effect on the phenotype of the adriamycin exposed embryos. We conducted independent series of experiments through the co-injection of the PACAP splicing morpholinos (*adcyap1a and adcyap1b*) or the human PACAP-38 peptide (5μM) and next exposed the different groups to either 0, 10 or 30 μM of adriamycin starting from 9 hpf until 57 hpf as previously described. Compared to the adriamycin only exposed embryos, the injection with PACAP morpholinos had a devastating effect on both the 10 and 30 μM adriamycin exposed embryos and the effect was progressive between 4 dpf and 6 dpf (Fig 8A and 8B). Interestingly, human PACAP-38 could rescue the toxic phenotype induced by both concentrations of adriamycin (Fig 8C).

## Discussion

The initial aim of this study was to generate a nephrotic syndrome animal model to evaluate the role of PACAP deficiency in the increased risk of arterial thrombosis that was detected in human NS patients. As our observations in humans showed more pronounced PACAP deficiency and increased platelet counts in CNS compared to INS [9], we decided to use a CNS animal model rather than a model mimicking minimal change nephrotic syndrome (MCNS) or focal segmental glomerulosclerosis (FSGS). As mentioned previously, transgenic CNS rodent models are not suitable because of early lethality after birth [28]. An endogenous nephrin knockout mouse model, with podocyte-specific, doxycycline-inducible expression of rat nephrin has later been developed [29]. Doxycycline-induced rat nephrin expression immediately after birth could rescue nephrin-deficient mice from perinatal lethality. This model shows proteinuria from the third week after birth, but considering the induced nephrin expression, it is not a pure CNS model. We used zebrafish CNS models for our study, as PACAP and ceruloplasmin levels, as well as thrombocyte counts, can be determined early after maturation of the pronephros in such models.

As a genetic nephrin knockout zebrafish model is not available, a first CNS zebrafish model was obtained using a nephrin targeting morpholino. Nephrin downregulation was confirmed in the nephrin morphant *Tg(cd41*:*EGF)* model, as previously described [21]. Distinct from our findings in human CNS patients [9], we did not observe PACAP peptide deficiency or increased thrombocyte numbers in these nephrin depleted embryos. On the other hand, when we tested the gene expression of both zebrafish PACAP genes in nephrin depleted embryos during the first 3 days of development we found a significant downregulation of *adcyap1a* but not *adcyap1b* compared to the controls (S2 Fig). Using whole mount in situ hybridization, Alexandre et al reported that both genes were expressed mainly in the brain and spinal cord during early development and that *adcyap1b* gene was higher expressed (≈4 folds) compared to the *adcyap1a* gene [30]. This might explain the lack of PACAP deficiency on the protein level in this model.

Another possible explanation is that the short duration of proteinuria with subsequent mortality of the embryos was not sufficient for developing PACAP and ceruloplasmin deficiency. Moreover, the phenotype of pericardial edema was achieved only in a small percentage of nephrin depleted embryos. A large percentage of embryos still had a normal phenotype at lower concentrations of injected nephrin morpholino, while high mortality was observed at higher concentrations.

The downregulation of both *adcyap1a* and *vip* genes in the nephrin depleted embryos might point to a shared perturbation of their activities in CNS; however, this perturbation doesn’t induce a palpable effect on thrombopoiesis in the zebrafish. It would be interesting to study the hemodynamics and the effects of VIP on thrombopoiesis and platelet function in human CNS patients, especially that the outcome effect is easily observable [9].

A second recently described CNS zebrafish model was also studied [22]. Adriamycin exposure suppresses nephrin expression and subsequently causes functional impairment of the glomerular filtration barrier, leading to proteinuria and pericardial edema early after fertilization, similar to human CNS. We examined the possibility of using adriamycin exposed *Tg(cd41*:*EGF)* zebrafish embryos as an alternative model to study the effect of PACAP on thrombocyte formation in NS. We confirmed nephrin depletion, impaired function of the glomerular filtration barrier, and a dose and time dependent increase of pericardial edema in adriamycin exposed embryos, as previously described [22]. At the RNA level, only *adcyap1a* was downregulated upon higher dose of adriamycin (30 μM) and relatively late at 6 dpf. Since PACAP is deficient at the protein level earlier in this model we think that inhibitory effects of adriamycin on peptide synthesis or the direct peptide loss through the kidney due to proteinuria may play the major role in this deficiency.

On the other hand, ceruloplasmin levels and GFP-labeled thrombocyte numbers remained unchanged with adriamycin exposure. We previously observed that children with INS have mildly decreased plasma PACAP levels, but maintain normal serum ceruloplasmin levels and demonstrate increased platelet counts only in a subgroup of the patients [9]. Hence, the adriamycin exposed zebrafish model seems to mimic the observations in human INS rather than CNS. We hypothesized that a longer duration of nephrosis might be necessary to affect megakaryopoiesis and platelet count. Furthermore, in humans with CNS, we observed low or normal platelet counts immediately after birth, slowly increasing within 1 or 2 weeks until they reached levels of 500–600 x 10^9^/L [9]. However, also for the adriamycin exposed zebrafish larvae that were analyzed at 5 and 6 dpf, they showed no change in ceruloplasmin levels or thrombocyte counts.

Adriamycin is known to be a cytotoxic drug. It increases oxidative stress and inhibits nucleic acid and protein synthesis of many essential enzymes and proteins [31]. Some of these might be involved in PACAP synthesis. So, although adriamycin causes nephrin downregulation and nephrotic syndrome in the zebrafish, it might not fully mimic the pathophysiologic state of the human NS.

Like other cytotoxic drugs, adriamycin can also cause myelosuppression in humans. Leukopenia is the most common, but moderate thrombocytopenia can also rarely occur and in a dose-dependent effect [32]. Hence, a direct inhibitory effect of adriamycin on thrombocyte formation in this zebrafish model cannot be excluded. A dose-dependent decrease of GFP-positive thrombocytes might then be expected and could counteract the effect of the demonstrated PACAP deficiency at this early stage.

As adriamycin can also cause cardiomyopathy in humans [33] and adult zebrafish [34], the question arose if the observed pericardial edema in the adriamycin exposed fish could be explained by cardiac toxicity, besides proteinuria. However, the cardiac phenotype in this model was previously studied in detail. Embryos exposed to low concentrations of adriamycin did not show any signs of cardiac toxicity or vascular abnormalities. Only higher concentrations (>40 μM) gave rise to morphological and functional cardiac changes and dysmorphic blood vessels. Moreover, dose-dependent proteinuria, associated with increasing cardiac edema was demonstrated, enforcing the causal relationship between proteinuria and edema in this model [22].

The explanation for the fact that our platelet findings for human CNS could not be replicated in these zebrafish CNS models, could be the important differences between thrombocyte formation in zebrafish and platelet formation in humans. The early process of hematopoiesis, including the formation of hematopoietic stem cells and megakaryocytes (or thrombocyte progenitors for the zebrafish), is comparable in both species [35]. However, zebrafish thrombocytes are very different from human platelets, as they remain nucleated [36]. The final maturation process from thrombocyte progenitor to thrombocyte seems to be different in zebrafish when compared to proplatelet formation from megakaryocytes in mammals. The existence of a zebrafish equivalent of such proplatelet-forming megakaryocyte that undergoes fragmentation of its cytoplasm to produce platelets has never been demonstrated. If PACAP deficiency exerts its effect during this late stage of megakaryopoiesis in humans [37], this could be another reason why our observations in humans were not reproducible in studied CNS zebrafish models.

One of the major findings of this study was revealing the renoprotective role of PACAP against adriamycin induced toxicity in the zebrafish. PACAP has previously been reported as protective against the cardiotoxic effects of adriamycin *in vitro* in rat cardiomyocyte culture and *in vivo* in mice [38,39]. It has also been reported to have renoprotective effects in murine models against multiple renal injuries as cisplatin-induced toxicity and contrast and diabetic nephropathies [40–42]. However, this is the first study that demonstrated a renoprotective effect of PACAP in the zebrafish. The fact that PACAP couldn’t rescue the phenotype of the nephrin morpholino further confirms our previous observation that adriamycin exerts its renal toxicity not only through nephrin downregulation but also through other mechanisms that can cause PACAP deficiency and therefore might be susceptible to PACAP therapy.

In conclusion, we established in this study two different CNS models in *Tg(cd41*:*EGFP)* transgenic zebrafish, by injection of a nephrin morpholino or exposure to adriamycin. In the nephrin depleted zebrafish model, neither PACAP deficiency nor increased thrombocyte counts were observed. PACAP deficiency was observed in the adriamycin exposed model; however, thrombocyte counts were not increased. We also confirmed that PACAP splicing morpholinos worsened the phenotype in both CNS models and that human PACAP-38 peptide could rescue the nephrotic phenotype in the adriamycin exposed model, but not in the nephrin depleted model. We conclude that none of the studied CNS larval zebrafish models were suitable for investigating the role of PACAP deficiency in megakaryopoiesis in CNS and that the adriamycin exposed model is a more suitable model to study PACAP deficiency and rescue in the zebrafish.

For future research, PACAP deficiency effect on megakaryopoiesis can be studied in other mammalian animal models of NS, such as the adriamycin treated mice [43]. On the other hand, the PACAP deficient zebrafish model reported in our study can be beneficial in the study of other physiological and pathological mechanisms involving PACAP in the zebrafish, such as brain development [44], energy homeostasis [45] and neuronal injury [46].

## Supporting information

S1 Fig Determining the PACAP-38 peptide rescue concentration. (A) Amino acid alignment between the human PACAP-38 and zebrafish PACAP-38. (B) Phenotype categorization after the injection of different concentrations of human PACAP-38 with *adcyap1a* and *adcyap1b* morpholinos (Minimum of 50 embryos were injected per condition).
(TIF)

S2 Fig Relative gene expression of *adcyap1a* and *adcyap1b* in the nephrin depleted (A,B) and the adriamycin exposed (C,D) CNS models. (TIF)

S3 Fig Relative gene expression of *vip* in the nephrin depleted (A) and the adriamycin exposed (B) CNS models. (TIF)
