# Supplementary figures and images for: Pituitary adenylate cyclase-activating polypeptide (PACAP) in zebrafish models of nephrotic syndrome
Source: PLoS One. 2017 Jul 31;12(7):e0182100. doi: 10.1371/journal.pone.0182100 (PMC5536324; doi:10.1371/journal.pone.0182100)

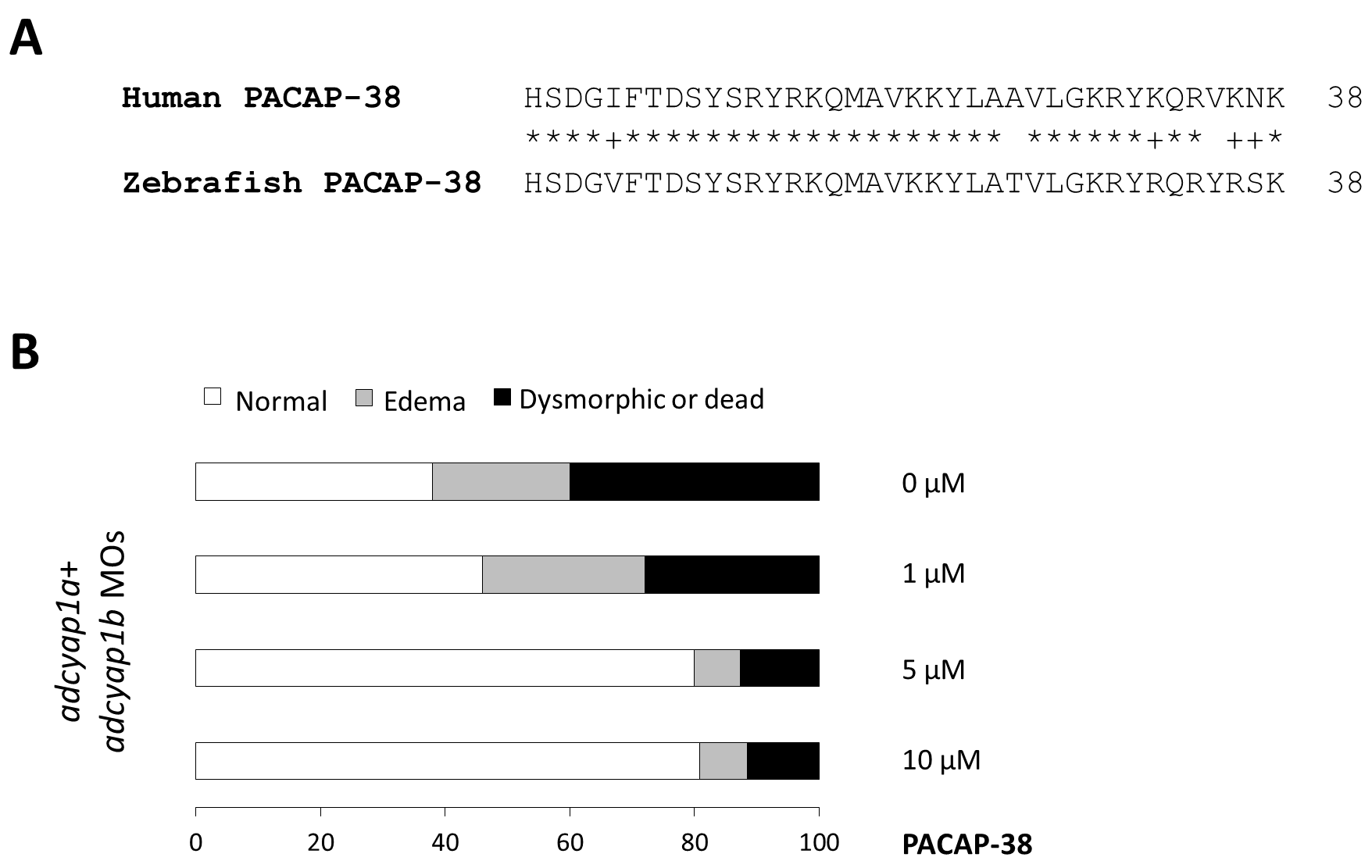

Supplement: S1 Fig — (A) Amino acid alignment between the human PACAP-38 and zebrafish PACAP-38. (B) Phenotype categorization after the injection of different concentrations of human PACAP-38 with adcyap1a and adcyap1b morpholinos (Minimum of 50 embryos were injected per condition). (TIF) [file pone.0182100.s001.tif]

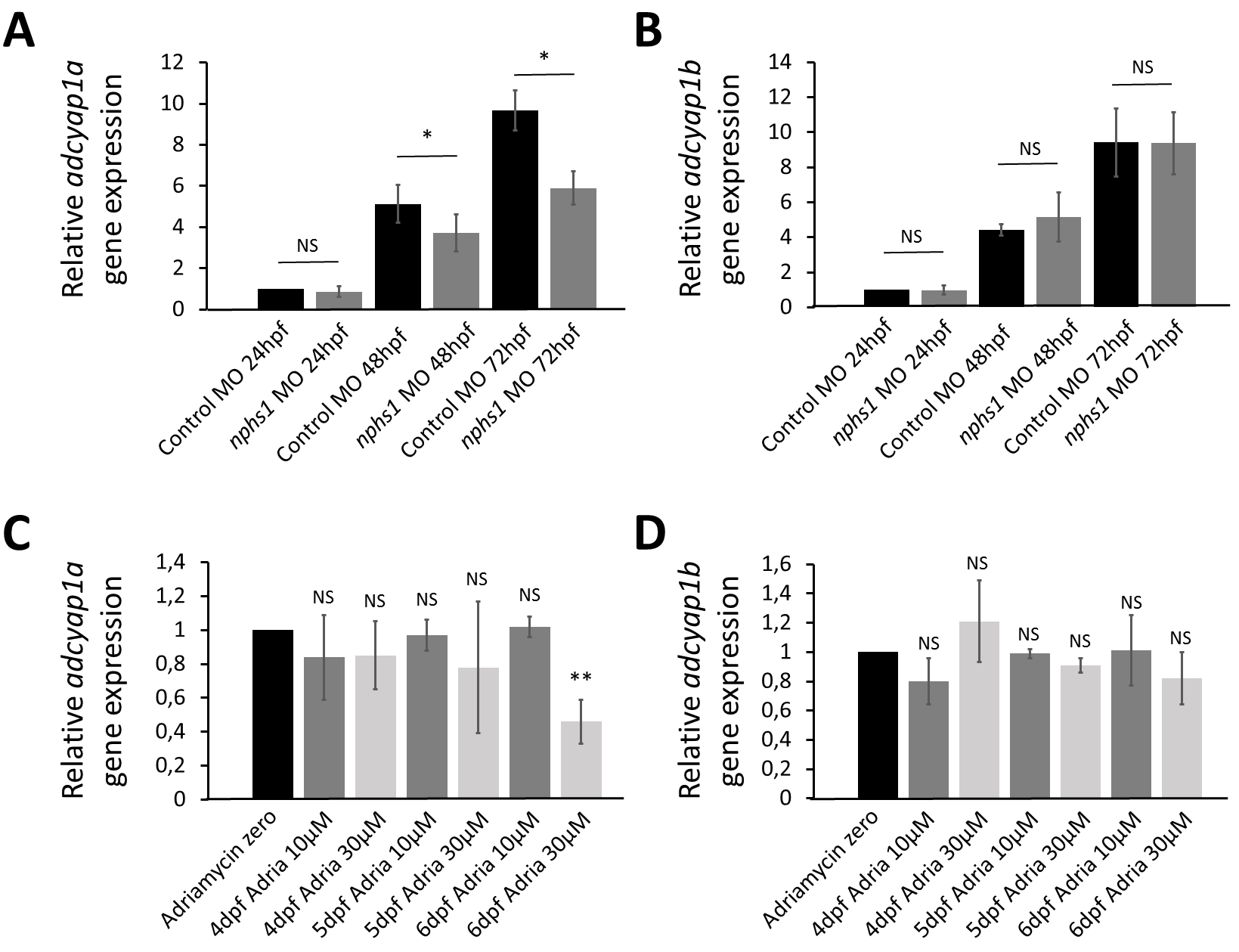

Supplement: S2 Fig — (TIF) [file pone.0182100.s002.tif]

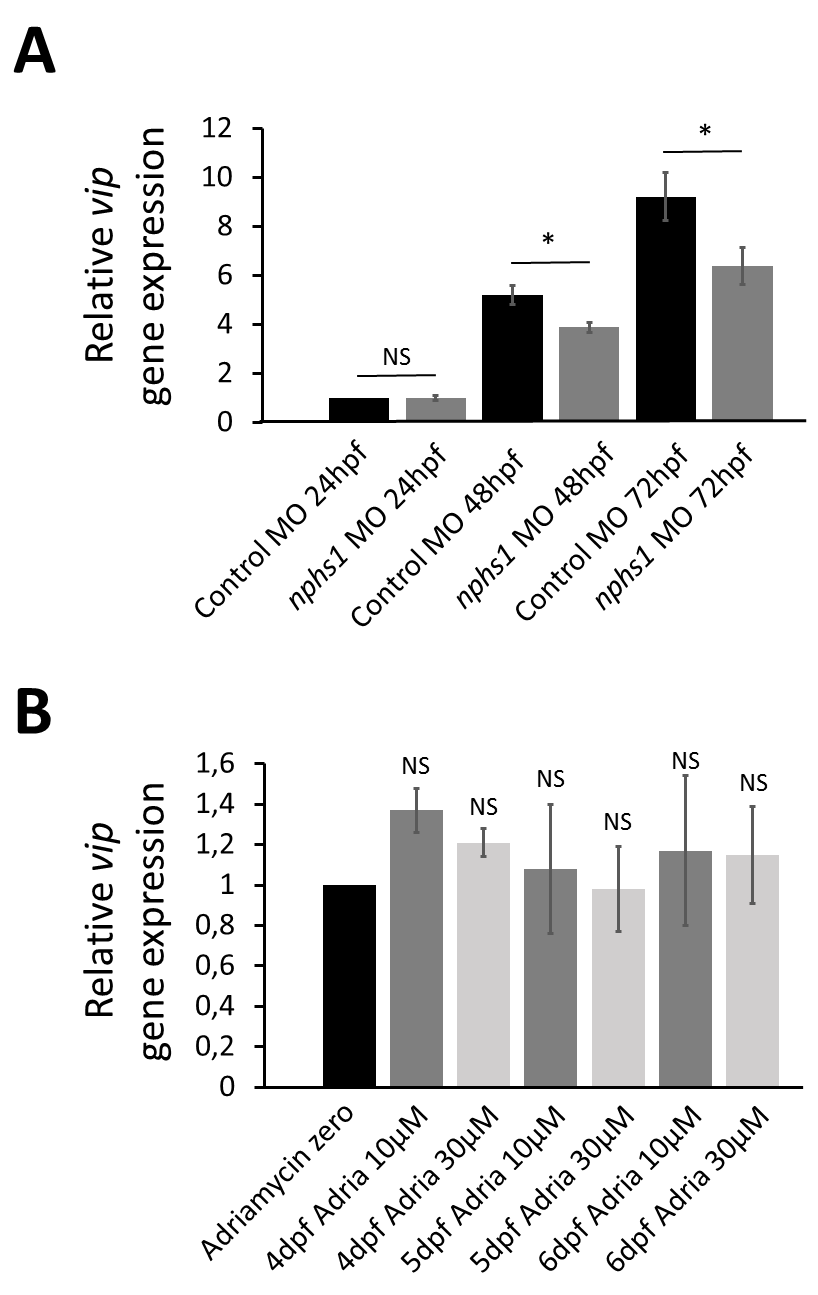

Supplement: S3 Fig — (TIF) [file pone.0182100.s003.tif]
